# The Effect of Hybrid Convective–Infrared–Ultrasonic Drying on Selected Properties of Kale and Nutritional Value of Kale Bars

**DOI:** 10.3390/molecules31152683

**Published:** 2026-07-31

**Authors:** Hanna Kowalska, Agnieszka Salamon, Jadwiga Wolska-Wilk, Małgorzata Chobot, Mariola Kozłowska, Klaudia Wieczorek, Agata Marzec, Jolanta Kowalska

**Affiliations:** 1 Department of Food Engineering, Warsaw University of Life Sciences, 159c Nowoursynowska St., 02-776 Warsaw, Poland; 2 Department of Grain Processing and Bakery, Institute of Agricultural and Food Biotechnology—State Research Institute, Rakowiecka 36 Street, 02-532 Warsaw, Poland; 3 Department of Chemistry, Warsaw University of Life Sciences, 159c Nowoursynowska St., 02-776 Warsaw, Poland; mariola_kozlowska@sggw.edu.pl

**Keywords:** vegetable snacks, vegetable bars, blanching, hybrid drying, freeze-drying, polyphenols, chlorophylls, antioxidant activity, fiber, minerals

## Abstract

The study aimed to determine the content of biocompounds and the nutrient composition of dried kale (with/without blanching) and bars containing it (fresh/dried). Kale leaves and bars were dried using convection (CD), convection–infrared–ultrasonic (HD), and freeze-drying (FD) methods to obtain potential snacks. The bar recipe consisted of 20% fresh kale (plus 30% water) or 10% dried kale (plus 40% water), 20% dried dates, 10% coconut flour, 10% flaxseed, and 10% hemp protein. Dried blanched kale contained from 524 to 618 mg of GAE/100 g d.m., which was 6.8–12.4% more than in unblanched kale, but about four times less in the bars. Similarly, blanched kale had a higher content of chlorophyll A + B and carotenoids. Both of these groups of compounds in blanched kale dried using the HD method were comparable to or slightly higher than in the raw material (approx. 932 and 174 mg/100 g d.m. of chlorophyll A + B and carotenoids, respectively), while in the bars their content was low, 34–64 and 7.6–13.2 mg/100 g d.m., respectively. The caloric content of the bars ranged from 325 to 348 kcal/100 g (1356 to 1452 kJ/100 g of product). Dried kale has proven to be a valuable snack due to its antioxidant content. In turn, the bars were rich in nutrients such as protein (approx. 14.4%), fat (approx. 9.3%), minerals (approx. 4.0%), carbohydrates (approx. 35.2%), and especially fiber (approx. 27.3%), which allows them to be classified as “high-fiber” products.

## 1. Introduction

Dried vegetable snacks, including bars produced using drying technology, are gaining importance as convenient foods because they provide concentrated sources of nutrients and bioactive compounds while facilitating vegetable consumption, which is still missing in human diets [1,2,3,4,5,6]. Their popularity is increasing due to growing consumer demand for healthier alternatives to conventional snacks, particularly products with high nutritional value, appealing sensory properties, and clean-label formulations. The possibility of including cereals, fruits, seeds, nuts, and vegetables, including secondary ingredients, but without unnecessary additives, makes plant-based bars characterized by high nutritional density; they are rich in dietary fiber and other bioactive compounds, and a properly developed formula can also provide a low glycemic index [4,7,8].

Kale (*Brassica oleracea* var. *acephala*) is a particularly promising raw material for dried snacks because of its high content of vitamin C, carotenoids, glucosinolates, flavonoids, and phenolic compounds [9,10,11,12], as well as dietary fiber, which does not limit calcium absorption [9,13]. Owing to its nutritional value and health-promoting properties, including potential benefits for the gut microbiome and the prevention of chronic diseases [14,15], kale has attracted growing interest as an ingredient in functional foods, such as dried vegetable products and snacks [11,15,16,17].

Pretreatments are commonly applied before vegetable drying to improve process efficiency and product quality. Steam blanching limits microbial growth and enzymatic browning while minimizing losses of vitamin C, chlorophyll, antioxidant activity, and color, and may also improve the bioavailability of selected bioactive compounds [18,19].

Drying is one of the key processes determining the shelf life, texture, nutritional value, and consumer acceptance of food products. However, it may induce undesirable changes [20], including non-enzymatic browning, oxidation, vitamin degradation, and protein denaturation, depending on the raw material and processing conditions. Consequently, increasing attention has been paid to hybrid drying technologies that combine different heat-transfer mechanisms, such as convection, infrared radiation, and microwaves, with supporting techniques, including ultrasound, to improve process efficiency while maintaining product quality [1,21,22,23,24]. Microwave-assisted drying accelerates moisture removal through volumetric heating but requires careful control of microwave power to avoid quality deterioration [25,26,27]. Ultrasound enhances mass transfer through acoustic cavitation and partial disruption of plant tissues, facilitating moisture migration and reducing drying time and energy consumption while improving the retention of bioactive compounds [23,28,29]. Infrared radiation further increases drying efficiency by rapidly heating the material surface and accelerating water evaporation, thereby shortening drying time and limiting thermal exposure. The combination of convection, infrared radiation, and ultrasound, therefore, offers considerable potential for improving drying efficiency while maintaining product quality.

Although the individual effects of convection, infrared radiation, and ultrasound on drying efficiency have been extensively studied, studies evaluating their combined use in kale drying and the impact on the quality of kale-based snack bars remain limited. Therefore, the synergistic effect of these mechanisms was expected to improve moisture removal while reducing thermal damage, allowing hybrid drying to achieve product quality comparable to freeze-drying but with greater practical potential. Therefore, this study aimed to evaluate the effects of blanching and different drying methods—convective drying (CD), hybrid convective–infrared–ultrasonic drying (HD), and freeze-drying (FD)—on the physicochemical properties and bioactive compound content of kale leaves. A further objective was to determine how the form of blanched kale, used either fresh or after drying, affected the nutritional value, physicochemical properties, and sensory quality of kale-enriched bars. It was hypothesized that hybrid convective–infrared–ultrasonic drying would preserve the nutritional and health-promoting properties of kale to an extent comparable to that of freeze-drying and enable the production of bars with desirable nutritional and sensory characteristics.

## 2. Results

### 2.1. The Influence of Blanching and Drying Method on Physicochemical Properties of Dried Kale and Bars

**Figure 1:**
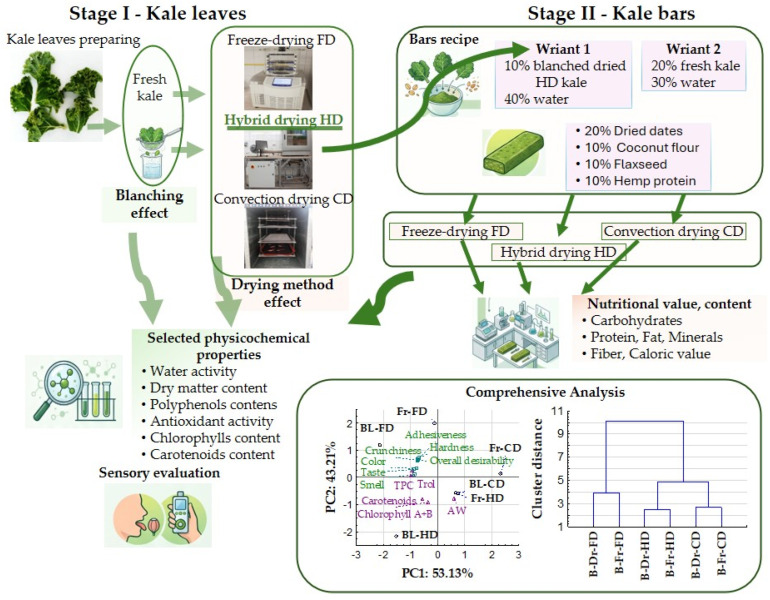
Schematic diagram of the experimental project.

The experimental design (Figure 1) examined the effect of kale blanching on selected physicochemical properties across the following drying methods: convection drying (CD), convection–infrared–ultrasonic drying (HD), and freeze-drying (FD). Similarly, for kale bars, the effects of the form of added kale (fresh or dried) and the above drying methods on the nutritional value and content of selected biocomponents were examined. To maintain the same proportions of fresh and dried kale leaves in the bars, the dry matter content was taken into account, and the appropriate amount of water was added.

#### 2.1.1. Dry Matter Content and Water Activity

**Figure 2:**
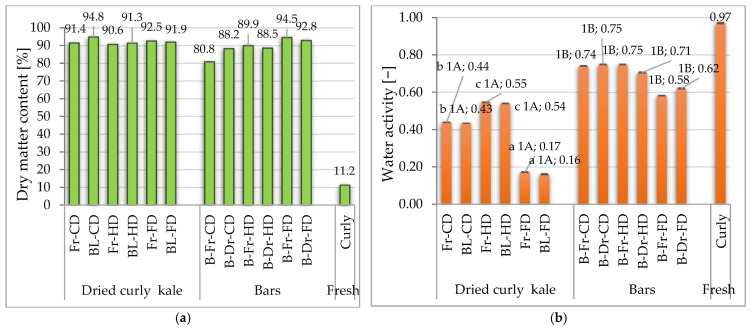
Dry matter content (**a**) and water activity (**b**) of kale leaves and kale bars. Designations: Fr—fresh kale, BL—blanching kale, CD—convection drying, HD—convection–infrared–ultrasonic drying, and FD—freeze-drying. Homogeneous groups for kale leaves: a–c—effect of drying method, 1A–1B—differences between the product (dried kale leaves, dried bars). No letters next to the values indicate no statistically significant differences at *p* = 0.05.

Drying kale allowed maximizing dry matter (DM) content, thereby minimizing moisture by concentrating ingredients and also obtaining dried snacks with a longer shelf life. In combination with water activity, it is a key parameter that determines the quality, stability, and functional value of the product, including its physicochemical and sensory properties. The drying method did not significantly affect the dry matter (DM) content of dried kale leaves and bars (*p* > 0.05) (Figure 2a). However, the DM values of kale (90.6–94.8%) were in a much narrower range than those of the bars (80.8–94.5%). While convectively dried kale showed slightly higher values than in the other samples, a tendency was observed for these values among the bars from convectively dried samples, convection–infrared–ultrasonic dried, and freeze-dried samples.

However, the water activity (AW) of dried leaves was influenced by the drying method (homogeneous groups a–c), and significant differences were observed between the dried products, kale, and bars (homogeneous groups 1A–1B) (Figure 2b). Higher dry matter content (DMC), ranging from 90.6% to 94.8%, was found in the leaves, while lower dry matter content (DMC), ranging from 80.8% to 94.5%, was found in the bars. This is understandable due to the drying efficiency of thin, slightly shredded leaves compared to bars with an initial dimension of 10 × 40 × 15 mm. In the study by Kowalska et al. [3], the DM content of multigrain bars with and without curly kale added, baked at 180 °C for 20 min, was lower, ranging from 59 to 73%. Therefore, it should be emphasized that in the case of bars, it is beneficial to maintain a softer structure, which is created by the higher product moisture. The drying method and blanching had no effect on the DM of kale, and similarly, in the case of bars, there was also no effect on the form of added curly kale and the drying method. Although freeze-drying yields higher DM values in most dried products than other methods, only bars dried this way achieved slightly higher values. In the case of kale leaves, DM values were more uniform, likely due to their thinness.

The drying method significantly affected the water activity of both products. Dried leaves exhibited an AW below the required value of 0.6, which corresponds to the limit below which a dried product is safe because microorganisms cannot grow. The lowest values were for leaves dried by freeze-drying (FD, below 0.2), and the highest for convection–infrared–ultrasonic drying (HD, up to 0.55). In the case of bars, similar AW values, ranging from 0.58 to 0.62 and visible in the graph, were only observed for freeze-dried products, while the remaining products were in the higher range of 0.71 to 0.75. Although most low-moisture products require water activity below 0.6, higher values are permissible for dried bars, provided they are packaged appropriately, use additional preservation methods, and have a shorter shelf life than dried products. The study by Kowalska et al. [5] analyzing the effect of fiber addition and production method of bars containing whole-grain oat flakes, pumpkin and sunflower seeds, flaxseed, and fiber preparations showed that the bars had high AW values, from 0.75 to 0.87, compared to 0.91 to 0.95 in previous studies [3], but mostly below the range in which pathogenic microorganisms develop. At the same time, they found that baked samples with an acceptably soft inner part of the bars had significantly higher water content and activity than convection–infrared–ultrasonic-dried bars.

#### 2.1.2. Chlorophyll A + B and Carotenoids Content

**Figure 3:**
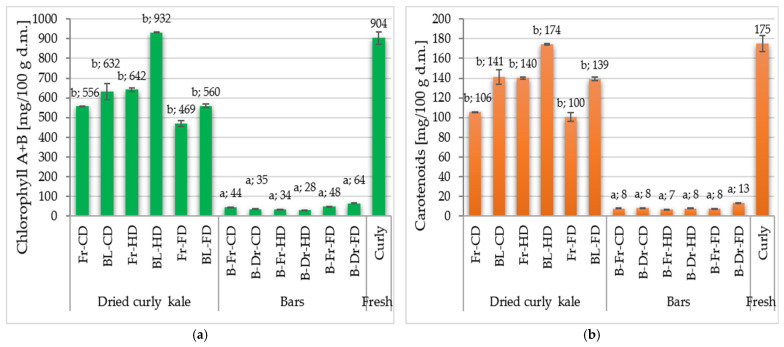
Chlorophyll A + B content (**a**) and carotenoid content (**b**) of kale leaves and kale bars. Designations: Fr—fresh kale, BL—blanching kale, CD—convection drying, HD—convection–infrared–ultrasonic drying, and FD—freeze-drying. Homogeneous groups for kale leaves: a–b—effect of drying method. No letters next to the values indicate no statistically significant differences at *p* = 0.05.

Chlorophyll, important in plant photosynthesis and as a natural pigment, obtained, for example, from plant waste, is used in the food, cosmetics, and pharmaceutical industries [30]. It is also a beneficial food ingredient and a visual indicator of product freshness. As a pigment, chlorophyll can impart a desirable green color to potential bars and also support health, including antioxidant and anti-inflammatory effects [31]. However, chlorophyll is very sensitive to processing and storage conditions, which can lead to degradation and loss of both color and bioactivity. Dried kale is rich in chlorophylls (469–932 mg/100 g d.m.) and carotenoids (100–174 mg/100 g d.m.) (Figure 3).

Unlike carotenoids, no significant effect of kale blanching and drying on chlorophyll content was observed. However, in both cases, drying blanched kale using the HD method resulted in the greatest preservation of these compounds, with levels similar to or even higher than those in the raw material. Furthermore, the FD method unexpectedly yielded the lowest levels of these compounds among the two methods. These results may be due to the drying duration, which was longest for freeze-drying (approx. 24 h). The use of convection–infrared–ultrasonic drying to support convective drying substantially shortens the drying time (approx. 1 h), thereby reducing kale’s exposure to elevated temperatures. This higher temperature could also have inactivated residual enzymes remaining after blanching, whose presence could have led to the degradation of chlorophylls and carotenoids when using the low-temperature FD method. Vargas et al. [32] demonstrated that kale dried by freeze-drying (FD) and refract window drying (RWD) exhibited less color change, indicating greater chlorophyll preservation than convection-dried samples. Chlorophyll is sensitive to various heat treatments, which can degrade it and other pigments.

Adding dried or fresh kale to the bars at 10 and 20%, respectively, although justified by the same dry matter content in the bar recipe, resulted in the presence of both types of compounds (chlorophylls and carotenoids) at amounts 10–20 times lower than those observed for dried kale leaves (Figure 3). Studies by Barakat and Almutairi [8] on date bars, in which dates were the main ingredient (40–60%) without kale, showed a higher content of carotenoids (approx. 327 mg/100 g). Korus [33] showed that the level of chlorophylls and carotenoids in 100 g of fresh kale leaves was approximately 121 mg of chlorophylls and 28.1 mg of carotenoids. However, their content in air-dried leaves increased to approximately 646 mg and 158 mg/100 g, respectively, and was 15% and 9% higher in freeze-dried leaves, respectively. Alasalvar et al. [34] reported that dried dates contain approximately 81 mg/100 g of carotenoids, primarily β-carotene, lutein, and zeaxanthin. Despite the concentration of these compounds during fruit drying, drying can lead to their loss or alteration of some compounds [34]. Their content in fresh fruits depends on the variety, growing conditions, and other factors [35].

#### 2.1.3. Polyphenol Content and Antioxidant Activity

**Figure 4:**
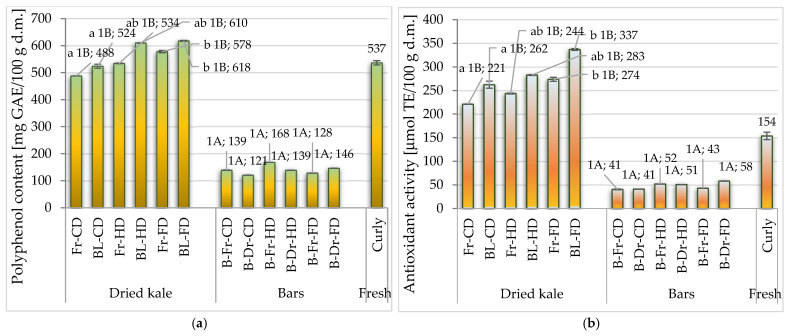
Polyphenol content (**a**) and (**b**)—antioxidant activity of kale leaves and kale bars. Designations: Fr—fresh kale, BL—blanching kale, CD—convection drying, HD—convection–infrared–ultrasonic drying, FD—freeze-drying. Homogeneous groups for kale leaves: a–b—effect of drying method, and 1A–1B—differences between products (dried kale leaves, dried bars). No letters next to the values indicate no statistically significant differences at *p* = 0.05.

Dried kale leaves contained significant amounts of polyphenols (488–618 mg GAE/100 g d.m.) (Figure 4a), exceeding the level in the raw material (approximately 537 mg GAE/100 g d.m.). The bars contained approximately 5-fold lower values of polyphenols (121–168 mg GAE/100 g d.m.) (Figure 4a). The drying method significantly affected their content. The highest values were obtained in kale dried using the FD method (average 598 mg GAE/100 g d.m.), and the lowest in the CD method (average 506 mg GAE/100 g d.m.).

Blanching caused a slight, several percent increase in kale’s polyphenol content. However, these values were significantly lower than those reported by Vargas et al. [32], who examined kale drying using hot-air drying, freeze-drying (FD), and refractance window drying (RWD). They showed that samples dried using the RWD method had the highest total phenolic content of approximately 18.8 mg/g (1877 mg/100 g) and flavonoid retention of 19.51 mg/g (1951 mg/100 g). In contrast, FD drying resulted in the lowest content of polyphenols (14.03 mg/g, i.e., 1403 mg/100 g) and flavonoids (18.72 mg/g, i.e., 1872 mg/100 g), but the highest content of glucosinolates and sulfur-containing compounds, which are abundant in cruciferous vegetables, including kale. Korus [36] demonstrated the benefits of freeze-drying kale, particularly when pre-blanching was used. Although it reduces the content of some minerals and vitamins, it significantly reduces nutrient losses, especially vitamins and tocopherols, during storage. According to Araújo et al. [18], steam blanching kale before convective drying was very beneficial for retaining vitamin C, total antioxidant capacity, and chlorophylls. However, the polyphenol content was retained by combining blanching with prior immersion in a metabisulfite solution. Blanching in these and our studies also had a positive effect on color and appearance parameters. Therefore, both fresh and dried kale are an excellent source of many desirable nutrients in the human diet [15]. Mierzwa & Szadzińska [25] reported that microwave-assisted convection drying of kale significantly reduces drying time but may negatively affect product quality. Therefore, it requires optimization of parameters, especially the duration of intermittent microwave exposure [25,26]. Dziki et al. [37] found that, regardless of the temperature in the 20–60 °C range, freeze-drying caused only a slight decrease in the total polyphenolic content and antioxidant activity, but a significant reduction in chlorophyll content compared to fresh leaves. Freeze-drying, considered the best food drying method, was suitable for drying kale due to its nutritional value, particularly vitamins and glucosinolates. As demonstrated in our research, convection–infrared–ultrasonic drying of kale leaves, especially blanched ones, also proved to be a good alternative.

While other ingredients in the bars could have influenced their levels, kale probably had a decisive influence on the polyphenol content in the bars. Given the great potential and benefits of kale, its use, including as an ingredient in value-added products, is limited [38]. Regardless of the drying method, the antioxidant activity (AA) of dried kale (221–337 μmol TE/100 g d.m.) was 1.4–2.2-fold higher than that of fresh kale (Figure 4b). FD drying resulted in the highest values (average 305 μmol TE/100 g d.m.), and the lowest values were obtained with the CD method (average 242 μmol TE/100 g d.m.). However, the antioxidant activity of the bars (41–58 μmol TE/100 g d.m.) was lower by nearly 6% and nearly 4% than in the dried kale. In the study by Barakat & Almutairi [8], date bars showed higher TPC, approximately 547 mg/100 g, as well as antioxidant activity (approx. 720 and 816 µmol TE/100 g, DPPH and ABTS, respectively).

#### 2.1.4. Nutritional Value of Selected Bars

**Figure 5:**
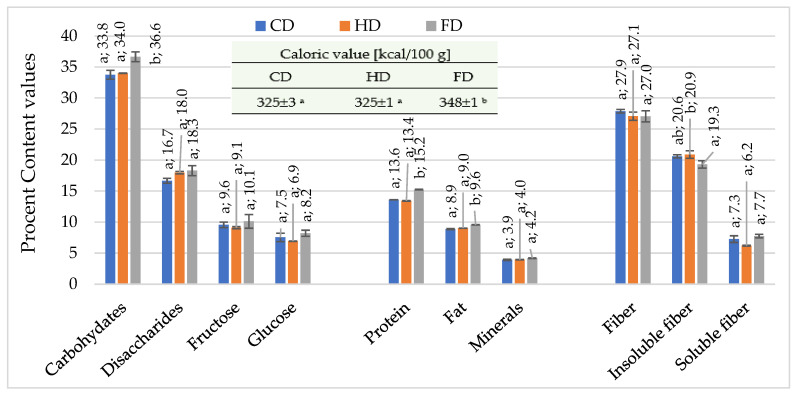
Nutritional value of bars containing 10% dried blanched kale. Designations: CD—convection drying, HD—convection–infrared–ultrasonic drying, FD—freeze-drying. Homogeneous groups for bars: a–b—effect of drying method at statistically significant differences, separately for each indicator, at *p* = 0.05.

In selected bars containing 10% dried blanched kale, the nutritional content was assessed, and the energy value of the bars was calculated (Figure 5). Among the nutrients, carbohydrates had the largest share in the bars, ranging from 33.8 to 36.6%, depending on the drying method (Figure 5).

The bars obtained by freeze-drying had the highest carbohydrate content, as well as protein, fat, and minerals, which was associated with the lowest water content, i.e., below 10% (Figure 2 and Figure 5). While freeze-dried bars had significantly higher total sugar content than bars from other drying methods, the drying method did not affect the content of disaccharides, fructose, or glucose. Disaccharides constituted the largest share of carbohydrates, ranging from 16.7 to 18.3%, while among monosaccharides, fructose had a larger share (9.1 to 10.1%) than glucose (6.9 to 8.2%). Compared to protein and fat content, the 3–4 times higher carbohydrate content had the greatest impact on the bars’ caloric value. Caloric content per 100 g of bars ranged from 325 to 348 kcal. Therefore, the bars can be classified as high-calorie snacks. However, it is worth emphasizing that they contain neither added sugars nor added fats. Their high nutritional value stems from the ingredients used. The main source of carbohydrates was dates, fat was linseed, and protein was hemp protein. The selected recipe resulted in a very high dietary fiber content (27.0–27.9%) in the bars, mainly in the water-insoluble form (19.3–20.6%). Both soluble and insoluble fiber play important physiological roles, contributing to gastrointestinal health, satiety, and metabolic regulation.

However, only the insoluble fiber forms showed a drying-method effect; those dried by HD had the highest content, while those dried by freeze-drying had the lowest. Both forms of fiber are useful in the human diet. According to Regulation (EC) No. 1924/2006 [39], food containing at least 6 g of dietary fiber per 100 g may be labeled as “high fiber”, while those containing 3 g/100 g qualify as a “source of fiber”. Ropero et al. [40] demonstrated that these nutrition claims are common in cereal-based products, including bars. Consuming one bar (~40 g) can provide approx. 44% of the recommended daily fiber intake, which is especially important considering that dietary fiber intake is still insufficient in many populations [41].

### 2.2. Sensory Evaluation

**Figure 6:**
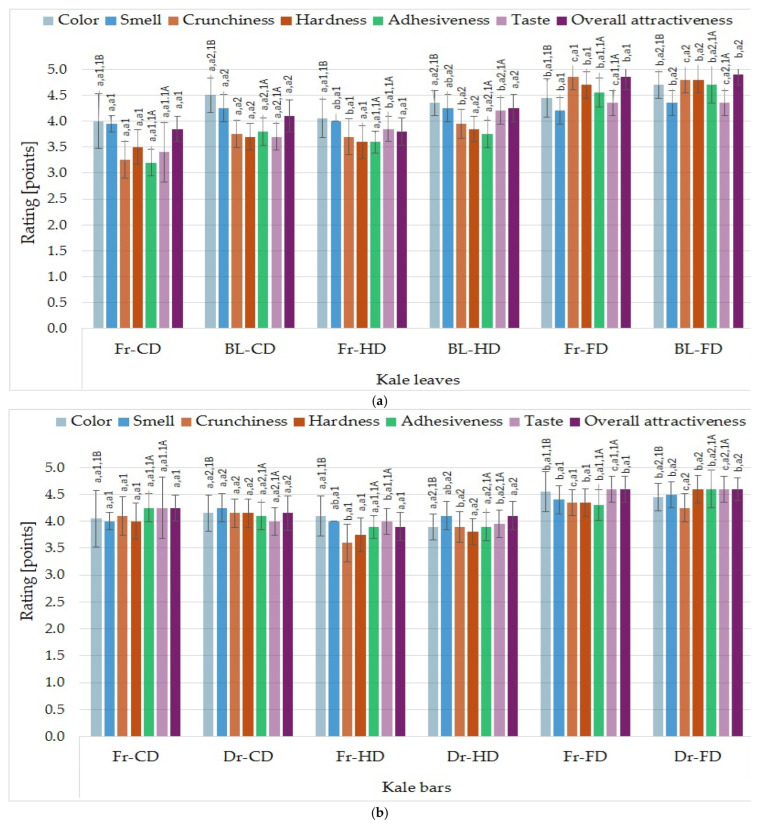
Sensory evaluation of kale leaves (**a**) and (**b**)—kale bars. Homogeneous groups for kale leaves: a–c—effect of drying method, a1–a2—effect of blanching; 1A–1B—differences between products (dried kale leaves, dried bars). No letters next to the values indicate no statistically significant differences at *p* = 0.05.

Both types of samples (dried kale and kale bars) received high ratings, particularly those obtained by freeze-drying (Figure 6). The mean values of all ratings ranged from 3.2 to 4.9 (on a 5-point scale) (Figure 6). Considering the mean values of all the attributes, the bars were rated slightly higher (from 4.1 to 4.3) than the kale (from 3.9 to 4.3). Statistical analysis of the color of the kale showed a significantly higher rating than the bars’ color, while the stickiness and taste were rated significantly higher for the bars. The significant differences between these ratings were only 3.2% to 6.1%. In terms of overall attractiveness on a 5-point scale, the FD-dried kale received a rating of 4.9, both with and without blanching. However, blanching resulted in significantly higher ratings for all the dehydrated kale characteristics. This was due to the attractive appearance, especially the color of the kale leaves, their preserved shape, delicate crunchiness, and hardness, as well as the overall attractiveness of the blanched samples. However, the form of kale added to the bars (fresh or dried) had no significant impact on any sensory characteristics, although, apart from color and overall attractiveness, samples with dried kale received slightly higher ratings than those with fresh kale.

For both products, the drying method had a significant impact on their sensory quality. All freeze-dried kale and bar samples received significantly higher ratings. The ratings for the hybrid dried kale, except for color, were intermediate between the highest ratings for the freeze-dried products and the lowest for the convection-dried ones. However, in most cases, the ratings for the HD samples were closer to the FD samples.

The lowest scores, in a similar range of 3.5–3.8, were obtained for kale samples dried using the hybrid method (3.7–3.8 points), and even lower scores for crunchiness, hardness, and stickiness (3.5–3.6 points). This may be due to the difficulty of chewing kale, given its higher moisture content and the presence of small stem fragments characteristic of this plant, which could contribute to lower crunchiness, greater hardness, and increased adhesiveness during chewing compared to freeze-dried samples. Similarly, for kale bars, the lowest scores (3.8–3.9 points) were observed for these sensory characteristics, but only for convection-dried samples, whereas hybrid-dried samples (4.1–4.2 points) received higher scores, more similar to the freeze-dried samples (4.3–4.5 points). Lower scores for the adhesiveness of the dried kale, resulting from difficulty biting, should not pose a problem, as the bars used shredded kale.

### 2.3. Comprehensive Analysis and Discussion

Among the many techniques used to produce bars [4,8], drying and supporting treatments appear promising; however, they are still not widely applied. This may be related to the relatively low popularity of vegetable snacks, particularly kale-based snacks and kale-containing snack bars. Therefore, further detailed research in this area represents an important research gap.

**Figure 7:**
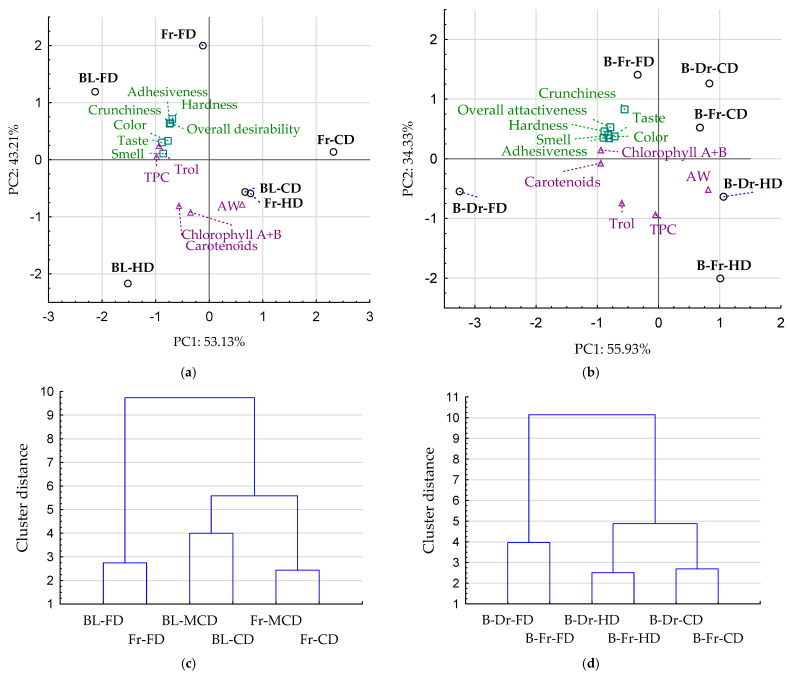
Principal component analysis (PCA) of physicochemical properties in correlation with the type of samples; (**a**)—biplot of kale leaves, and (**b**)—biplot of kale-bars, (**c**,**d**)—cluster analysis of kale leaves and kale-bars, respectively. Designations: Fr—fresh kale, B—bar, BL—blanching, CD—convection drying, HD—convection–infrared–ultrasonic drying, FD—freeze-drying; Quality indicators: AW—water activity, Trol—antioxidant activity, TPC—total polyphenol content.

Considering the large number of variables and quality indicators associated with dried kale and kale-based bars, including pretreatment and drying conditions, principal component analysis (PCA) was used to interpret the complex relationships between the parameters studied. Figure 7a shows the PCA for dried kale, while Figure 7b shows the PCA analysis for the bars. PC1 and PC2 components explained 96.34% and 90.26% of the total variance for dried kale and kale bars, respectively. Biplot distributions of indicators related to bioactive compounds, water activity, and sensory evaluation showed similar patterns for both products. In both cases, samples obtained by freeze-drying (FD) were clearly separated from samples dried by the other drying methods. These relationships were further confirmed by cluster analyses presented in Figure 7c,d.

PCA results confirmed the beneficial effect of thermal pretreatment (blanching) on preserving desirable biocomponents in kale leaves (Figure 3, Figure 4 and Figure 7). Although FD provided the best overall quality protection, the highest chlorophyll and carotenoid contents were observed in blanched kale dried using the hybrid convection–infrared–ultrasonic (BL-HD) method. Unblanched samples dried at high temperature (Fr-HD), along with blanched samples dried by convection and freeze-drying (BL-CD and BL-FD), also exhibited high pigment contents. Consequently, these samples were associated with the same PC2 component and formed a separate cluster in the PCA biplot (Figure 7a). The different effects of freeze-drying and hybrid drying on phenolic compounds and photosynthetic pigments may be associated with differences in their chemical stability, cellular location, and extractability. The relatively high total phenolic content of freeze-dried kale can be attributed to the low-temperature and reduced-oxygen conditions of the process, which limit thermal and oxidative degradation. In the HD samples, comparable phenolic content may have resulted from a balance between partial degradation and enhanced extractability due to disruption of plant tissue and the release of phenolic compounds associated with the cellular matrix. Therefore, the measured total phenolic content may reflect not only the actual preservation of these compounds but also their accessibility during extraction. In contrast, chlorophylls and carotenoids are lipophilic photosynthetic pigments that undergo degradation through mechanisms different from those affecting phenolic compounds.

Chlorophyll degradation may involve the displacement of the central Mg^2+^ ion and the formation of pheophytins, particularly under acidic and thermal conditions [42]. Carotenoids may undergo oxidative degradation and cis–trans isomerization as a result of exposure to oxygen, light, and elevated temperature. The higher pigment contents obtained after HD may therefore be associated with rapid moisture removal and the shorter duration of the hybrid process, which limited the cumulative exposure of the pigments to degrading conditions. Freeze-drying, although generally effective in preserving thermolabile compounds, does not invariably provide the highest retention of photosynthetic pigments. The freezing and sublimation stages produce a highly porous structure that may increase pigment exposure to oxygen and light during subsequent handling, grinding, and extraction [43]. Di Cesare et al. [44] similarly reported higher chlorophyll retention after microwave drying than after freeze-drying of basil. The decrease in chlorophyll content during freeze-drying may also be related to residual chlorophyllase activity, as this enzyme plays a key role in chlorophyll degradation [45]. Because freeze-drying occurs at low temperatures, it does not guarantee complete enzyme inactivation, especially for enzymes that remain active after blanching. Consequently, insufficient blanching may allow chlorophyllase to remain in an active or potentially activatable state [46]. Upon rehydration or moisture absorption during storage, chlorophyllase can hydrolyze chlorophyll to chlorophyll, thereby contributing to further pigment degradation [45,47]. Higher temperatures used during HD may more effectively reduce residual enzyme activity, while shorter drying times and ultrasound assistance may limit exposure of pigments to conditions conducive to degradation. However, because pigment degradation pathways and structural changes were not directly examined in the present study, these mechanisms should be considered plausible explanations for the observed results.

Freeze-drying or convective–infrared–ultrasonic drying of bars preserved bioactive compounds more effectively than convective drying, resulting in the distinct clusters in Figure 7d. Ultrasound (US) induces two main phenomena in plant tissues: the so-called “sponge effect,” consisting of alternating cell compression and expansion, and acoustic cavitation, associated with the formation, growth, and implosion of gas bubbles. These mechanisms modify the tissue microstructure by creating microchannels, increasing the porosity and permeability of cell walls, and facilitating mass transport. The intensity of these changes depends on sonication parameters, such as frequency, power, and exposure time, as well as the properties of the raw material. Therefore, improperly selected conditions can lead either to favorable process intensification or to excessive structural damage and loss of valuable bioactive compounds [48]. Nowacka et al. [49] demonstrated that extended ultrasonic treatment of red beets improved some technological properties but increased betalain losses, emphasizing the need for process optimization. During infrared drying, IR radiation is absorbed by water and other components and converted into heat through changes in atomic bond vibrations, which increases the product temperature [50]. Consequently, infrared heating accelerates moisture evaporation and shortens drying time compared to conventional convective drying. Therefore, for both ultrasonic and infrared technologies, process efficiency and product quality largely depend on the appropriate selection of processing parameters [48,49].

**Figure 8:**
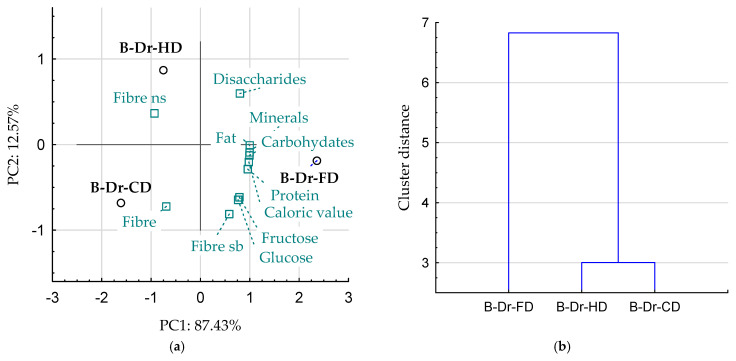
Principal component analysis (PCA) of nutritional value of dried bars; (**a**) biplot and (**b**) cluster analysis. Designations: B—bar, CD—convection drying, HD—convection–infrared–ultrasonic drying, FD—freeze-drying, Fiber ns—insoluble fiber fraction, Fiber sb—soluble fiber fraction.

Regardless of the product type, the chlorophyll content showed a strong positive correlation with the carotenoid content (0.82–0.90). Similarly, the correlation between polyphenols and antioxidant activity was high for dried kale (0.90), although lower for bars (0.63). Moreover, higher chlorophyll content was associated with better sensory evaluation across all assessed features (0.64–0.97). Carotenoids also had a positive effect on sensory characteristics, especially smell, hardness, adhesiveness, and overall attractiveness (0.62–0.81). PCA of the nutritional value of kale bars confirmed that the freeze-dried bars had the highest content of carbohydrates, proteins, and fats. Consequently, these components were strongly positively correlated with caloric value, and the FD samples formed a separate cluster (Figure 5 and Figure 8).

Freeze-drying is widely considered one of the best methods for producing high-quality dried foods while maintaining nutritional and sensory properties [51,52]. Supporting pretreatments can significantly reduce drying times, which remains one of the main limitations of this technology [53]. Convection–infrared–ultrasonic-dried bars showed intermediate values for most nutritional parameters, but were statistically assigned to the same homogeneous group as the convection-dried samples, both located on the negative side of PC1. This group was associated with, among other things, the highest insoluble dietary fiber content in these samples. Insoluble fiber showed a very strong negative correlation with caloric value (−0.99) and a weaker negative correlation with total dietary fiber (−0.53), and soluble fiber showed a positive correlation (0.74) with caloric value. The total dietary fiber content of the bars (27.0–27.9%) significantly exceeded the minimum requirement for a “high fiber” claim [39]. In addition to its nutritional value, dietary fiber contributes to water binding, structure formation, and sensory properties, making it an important ingredient in functional food [3].

While freeze-dried kale exhibited the best overall quality, hybrid drying also demonstrated significant potential when combined with infrared heating and ultrasound. Kale blanched and dried using this method contained approximately 66 and 25% higher chlorophyll and carotenoid content, respectively, than freeze-dried samples (Figure 3), comparable polyphenol content, and approximately 19% lower antioxidant activity (Figure 4). These results highlight the potential of hybrid drying for producing vegetable snacks and value-added food ingredients [38].

The lower concentrations of bioactive compounds observed in the bars compared to dried kale were most likely related to the lower proportion of kale in the overall formulation. Direct comparison with previous studies is difficult because comparable reports are currently not available. Nevertheless, the nutritional value of the developed bars deserves attention, as they were made exclusively from natural ingredients, including kale, dates, coconut flour, flaxseed, and hemp protein. The bars contained high amounts of protein (13.4–15.2%), fat (8.9–9.6%), minerals (3.9–4.2%), and carbohydrates, particularly dietary fiber, primarily its insoluble fraction. The average dietary fiber content of 27.3 g/100 g (approximately 8.3 g/100 kcal) exceeded the minimum level required for a “high fiber” claim by more than four times [39], while a 40 g serving provided approximately 10.9 g of dietary fiber, corresponding to about 44% of the adequate daily intake of 25 g established by EFSA for adults. Therefore, manufacturers can use nutritional claims on the packaging of such bars. These observations are consistent with previous studies using similar ingredients in bar formulations. Leonard et al. [54] demonstrated that hemp seed protein has high nutritional value and digestibility, while hemp protein concentrate and isolate contain 70–90% *w*/*w* protein. Israelsen et al. [55] reported that hemp protein was suitable for bars containing dates and coconut flour, resulting in a protein content of approximately 19.2%. It was also shown that coconut flour, flaxseed, and hemp protein primarily determine the fiber level (7–13%), while dates provide natural sugars (45–60%) and largely determine the energy value. Ahmad et al. [56] demonstrated that including 5–15% flaxseed in cookies and cereal bars increased protein content while maintaining favorable sensory acceptability. Kowalska et al. [5] found that cereal ingredients and fiber preparations influenced the nutritional value, energy content, sensory properties, and microbiological quality of dried and baked bars. In addition to oat flakes, pumpkin seeds, sunflower seeds, and flaxseed, they included fiber preparations such as psyllium fiber and suggested using byproducts such as apple pomace. Similarly, the bars developed in the present study contained moderate-to-high levels of fat (8.9–9.6%), derived primarily from the natural oils in hemp seeds and flaxseed [54,56], with coconut flour contributing to a lesser extent.

Overall, hemp protein and flaxseed effectively increased the protein and fiber content, while dates provided natural carbohydrates and sweetness in the bar recipe. Therefore, the developed bars represent promising plant-based snacks characterized by high dietary fiber content and a substantial protein contribution. These results indicate the need for further research to better understand the mechanisms that influence bioactive compound retention and the nutritional value of dried products, as well as to assess the broader potential of kale as an ingredient in vegetable snacks and functional bars. Given the growing consumer interest in plant-based products with enhanced nutritional value, the current findings provide a foundation for further development and optimization of these products.

## 3. Materials and Methods

### 3.1. Material and Technological Methods

The basic material for the work was kale purchased from a local supplier. Kale leaves of similar size were selected, washed, and dried on filter paper; thicker stems were discarded, and the leaves intended for testing were divided into rectangles measuring approximately 60 × 40 mm. Part of it was subjected to pretreatment, i.e., steam blanching for 60 s over boiling water (approx. 100 °C). Small portions of kale, approximately 30 g, were placed in a covered sieve and shaken lightly every 20 s.

Two bar variants were prepared, one with 20% fresh kale and 30% water, and the other with 10% dried kale and 40% water. The remaining ingredients for the bar mixture were 20% dried dates (EnerBio, Burgwedel, Germany) and 10% each of coconut flour and hemp protein (Purella, Warsaw, Poland), and flaxseed (Zdrowe Pola, Łódź, Poland). The dates and kale were ground using a Bosch grinder. After adding the ingredients, except for the water, the mixture was ground again for 60 s. Water was then added, mixed, and formed into 10 mm × 40 mm × 15 mm bars, which were then dried. The final bars weighed approximately 40 g each.

The three drying methods were applied to fresh and blanched kale leaves and to appropriately formed bars with the addition of dried kale, after or without blanching (fresh).

#### 3.1.1. Convection Drying

Fresh and blanched kale samples weighing approximately 150 g were placed on trays and dried at 60 °C in a laboratory convection dryer with forced airflow (approximately 2.0 m/s). Bars weighing approximately 150 g, formed in molds to a size of 40 × 110 × 15 mm, were dried under the same conditions. The process continued until stable products were obtained, i.e., until the kale reached a constant mass, and the bars reached water activity of less than 0.6, for approximately 3.5 h.

#### 3.1.2. Convection–Infrared–Ultrasonic Drying (Hybrid Drying, HD)

Material samples (raw and blanched kale, bars) were placed on a mesh tray allowing hot air flow in the chamber of a hybrid convection–infrared–ultrasonic dryer from PROMISE-TECH Inc. (Wrocław, Poland). Drying was carried out in four cycles under the conditions presented in Table 1. During drying, mass change was measured every 5 min. The drying process was repeated in the specified cycles and terminated when the mass of the dried product remained constant and the required water activity was achieved.

#### 3.1.3. Freeze-Drying

Before the freeze-drying process, the samples, weighed and vacuum-sealed in PA/PE foil, were placed on shelves in an shock freezer (HCM 51.20, Irinox, Treviso, Italy) with forced air circulation at a temperature of −40 °C for 24 h. Then, the samples were removed from the packaging and placed on trays in a freeze-dryer (Osterode am Harz, Germany) and dried for 24 h at a shelf temperature of 20 °C and a pressure of 63 Pa.

### 3.2. Analytical Methods

Water/dry solids content was determined by the gravimetric method (reference method) after drying at 105 °C according to AOAC 920.15, 2002 [57]. The method involves drying a weighed sample and comparing the sample mass before and after drying to a constant mass at 105 °C. Water activity was measured using an AQUALAB CX-2 device (Decagon Devices Inc., Pullman, WA, USA) at 25 ± 1 °C. These determinations were performed in duplicate.

Chemical determinations were performed in the accredited laboratory of the Institute of Agricultural and Food Biotechnology—National Research Institute in Warsaw. All determinations were performed at least twice. The assay procedures were based on the methodology described by Kowalska et al. [3].

Extract Preparation. Approximately 1.0 g of ground sample, depending on the sample type, was weighed into 50 mL Falcon tubes using a Sartorius Competence CP 224S-OCE analytical balance (Sartorius, Göttingen, Germany) with an accuracy of ±0.0001 g. Then, 25 mL of 80% acetone solution was added. The samples were homogenized in an ULTRA-TURRAX T25 basic homogenizer (IKA-WERKE, Staufen, Germany) for 30 s at 13,500 rpm. The homogenate was stored at room temperature in the dark for 4 h. The homogenate was then centrifuged in a laboratory centrifuge MPW 375 (MPW-Med-Instruments, Warsaw, Poland) for 3 min at 1000 rpm.

Carotenoid and chlorophyll content were determined using a BECKMAN DU-530 spectrophotometer (Beckman, Amersham, UK) [3]. The measurements were made for chlorophyll A at wavelengths λ = 663 nm, for chlorophyll B at λ = 647 nm, and at λ = 470 nm for carotenoids with the blank, which was an 80% (*v*/*v*) acetone solution. When the measured absorbance of the sample was greater than 0.900 in value, the sample was diluted with an 80% (*v*/*v*) acetone solution. The determination was performed in duplicate. The content of chlorophyll or carotenoid dyes in the sample was calculated in mg per 100 g dry matter (d.m.).

Total polyphenol content (TPC) was determined spectrophotometrically using the Folin–Ciocalteu reagent [3]. To the test tube were added 15% sodium carbonate (0.5 mL), distilled water (8.9 mL), acetone extract of the sample (0.5 mL), and 100 μL of Folin–Ciocalteu reagent. After mixing and incubating for 45 min in the dark (at room temperature), the absorbance was measured at λ = 765 nm against a blank. The sample was diluted with 80% (*v*/*v*) acetone solution when the measured absorbance was greater than 0.650. Total polyphenol content was expressed as mg of gallic acid equivalent per 100 g of dry matter (mg GAE/100 g d.m.).

Antioxidant activity (AA) was determined spectrophotometrically at a wavelength of λ = 515 nm [3]. Samples were prepared using 2.4 mL of a 60 μM methanolic DPPH radical solution and 100 μL of acetone extract. The samples were mixed and incubated at room temperature for 30 min in the dark. Absorbance against the blank was then measured. A solution of acetone and DPPH was used as a control sample. The blank contained methanol and 80% acetone. The antioxidant activity (AA), based on the extract’s DPPH free radical-scavenging ability, was expressed as µmol Trolox equivalent per 100 g of dry matter (µmol TE/100 g d.m.).

Protein content was determined based on the total nitrogen content determined by reference titration (Kjeldahl) and converted to total protein content, considering the nitrogen-to-protein conversion factor of 6.25 in accordance with the PN-EN ISO 20483:2014 standard [58].

Fat content was determined according to the PN-A-79011-4:1998 standard [59] by extracting fat from the dry sample using petroleum ether in a Soxhlet apparatus, then weighing the remaining sample after complete evaporation of the solvent.

Ash content was determined gravimetrically after incinerating the samples in accordance with the PN-EN ISO 2171:2023 standard [60] by incinerating the dry sample at 900 °C and determining the inorganic residue by gravimetry. Total dietary fiber content, including soluble and insoluble fractions, was determined gravimetrically after enzymatic hydrolysis of the samples using the Megazyme Total Dietary Fiber Kit (Bray, Bray Business Park, Co. Wicklow, A98 YV29, Ireland).

For sugar determination, ground (approximately 0.5 g) bar samples were extracted with 25 mL of distilled water at 25 °C for 3 h, centrifuged (6500 rpm, 5 min), filtered through a 0.45 μm syringe filter (Macherey-Nagel, Düren, Germany), and analyzed by HPLC-RI according to Ignaczak et al. 2024 [61]. Sugar separation was performed using a Waters Sugar-Pak I column (6.5 × 300 mm, 10 μm; Waters, Milford, MA, USA) maintained at 90 °C, with an RI detector (Waters 2414, Milford, MA, USA) set at 35 °C. The mobile phase consisted of 0.1 mM calcium disodium EDTA, fed isocratically at a rate of 0.5 mL/min; the injection volume was 10 μL, and the analysis time was 20 min. Sugars were quantified using calibration curves prepared from sucrose, glucose, and fructose standards.

### 3.3. Sensory Evaluation of Kale Snacks and Bars

The sensory evaluation was conducted by a trained team of 10 people (aged 18–52), including faculty members teaching sensory analysis and students from the Department of Food Engineering at the Warsaw University of Life Sciences (WULS). The students completed the sensory evaluation training and passed the relevant exam. The study was conducted as an analytical sensory evaluation to detect differences among the tested samples, following the methodology described by Ignaczak et al. [26].

Samples of dried kale and a separate batch of kale bars were placed in coded disposable cups and evaluated anonymously. The samples were rated on a five-point scale, including color, smell, crunchiness, hardness, adhesiveness, taste, and overall attractiveness. Definitions of the assessed sensory attributes and their threshold values are presented in Table 2. Each participant received fresh, still water to rinse their mouth between evaluations. Before the evaluation, panel members were briefed on the research procedure, sample characteristics, evaluation criteria, and planned use of the collected data. All participants gave verbal consent to voluntarily participate in the sensory evaluation and were informed of their right to withdraw from the study at any stage. The study did not require formal approval from an ethics committee. The rights and privacy of participants were protected in accordance with Regulation (EU) 2016/679 [62].

### 3.4. Statistical Analysis

Two series of tests were performed, and individual determinations were carried out in two repetitions. The results were statistically analyzed using Statistica 13 PL software (StatSoft, Krakow, Poland) and MS Excel 10. Analysis of variance (ANOVA) at a significance level of 0.05 was used to examine the effects of blanching and drying method on kale leaves, and, for the bars, the form of added kale (fresh, dried) and the drying method. Tukey’s HSD test was used to identify homogeneous groups. Pearson correlation and principal component analysis (PCA) were used to examine relationships between indicators. Similarities among data groups and samples were visualized using biplots and Ward’s clustering. Data were presented as means ± standard deviation.

## 4. Conclusions

Blanching kale leaves before drying can be considered an effective pretreatment because it increases water removal and reduces water activity, especially in freeze-dried samples, and also improves sensory quality and preserves selected bioactive compounds.

The drying methods used enabled the production of kale-based snacks with acceptable sensory properties and reduced water activity, suggesting their potential for obtaining products with extended shelf life. Freeze-drying ensured the highest overall preservation of nutritional and sensory quality, particularly with respect to polyphenols, antioxidant activity, and selected nutrients. The hypothesis that convective–infrared–ultrasonic (hybrid) drying could achieve product quality comparable to freeze-drying was largely confirmed, as this method produced products with high sensory acceptability and health-promoting potential, preserving chlorophylls and carotenoids more effectively than freeze-drying.

The optimal drying method depends on the target quality characteristics. While freeze-drying remains preferred when maximum preservation of overall nutritional quality is required, convective infrared ultrasonic drying offers a promising alternative, especially when preservation of pigments, especially chlorophylls and carotenoids, is essential, as is the development of more technologically accessible processes. Despite lower product quality, conventional convective drying remains a significant reference method due to its simplicity, operational reliability, and reduced technological complexity.

The observed relationships between chlorophylls, carotenoids, polyphenols, antioxidant activity, and sensory characteristics emphasize the importance of considering multiple quality indicators when evaluating drying technologies. The findings should be interpreted within the scope of the experimental conditions applied, including the specific kale material, blanching pretreatment, product formulation, and drying methods. The present study was limited to the analysis of selected nutritional and bioactive compounds and did not include a more comprehensive chemical or structural characterization. Another limitation is the lack of analysis of glucosinolates, despite their importance as characteristic bioactive compounds in kale. Therefore, future studies should expand the scope of analyses by including qualitative and quantitative determination of glucosinolates, broader chemical profiling, detailed microstructural characterization, investigations of transformation mechanisms and bioavailability of bioactive compounds, as well as techno-economic assessments to support the industrial application of kale-based functional snacks.

## Figures and Tables

**Table 1:** Parameters of convection–infrared–ultrasonic drying (HD).

Parameters	Cycle 1	Cycle 2	Cycle 3	Cycle 4
Time [min]	15	15	15	5
Air speed [m/s]	5	2	3	2
Infrared power [W]	250	0	250	0
Air temperature [°C]	80	20	80	20
Distance of infrared radiators from the material surface [cm]	25	25	25	25
Ultrasonic power [W]/frequency [kHz]	200/36	0	200/36	0

**Table 2:** Definitions and explanations of sensory attributes.

Sensory Feature	Definition	Point Scale	
		Dried Kale (Points)	Bars (Points)
Color	Color and color saturation of the bars	1—undesirable, uneven coloring 5—desirable, even, intense	1—undesirable, uneven coloring 5—desirable, even, and intensified
Smell	The intensity and attractiveness of the perceived scent	1—undesirable or imperceptible 5—desirable, perceptible	1—undesirable or imperceptible 5—desirable, perceptible
Crunchiness	Brittle, easily breaks under slight force. Rubbery is a plastic material	1—undesirable, rubbery 5—desirable, crunchy	1—undesirable, rubbery 5—desirable, crumbly
Hardness	Hard requires a lot of force to squeeze, while soft requires little force	1—very hard 5—very soft	1—very hard 5—very soft
Adhesiveness	The degree of particle adhesion to teeth	1—large 5—small	1—large 5—small
Taste	Felt after biting and/or chewing	1—undesirable, bitter, earthy 5—desirable, delicate	1—undesirable, bitter, earthy, sandy 5—desirable, slightly sweet
Overall attractiveness	Overall feeling (all characteristics), level of satisfaction	1—very poor 5—very good	1—very poor 5—very good

## Data Availability

The data presented in this study is available on request from the corresponding author.
